# Species Diversity and Resource Status of Macrofungi in Beijing: Insights from Natural and Urban Habitats

**DOI:** 10.3390/jof11080607

**Published:** 2025-08-21

**Authors:** Dong-Mei Liu, Shi-Hui Wang, Ke Wang, Jia-Xin Li, Wen-Qiang Yang, Xi-Xi Han, Bin Cao, Shuang-Hui He, Wei-Wei Liu, Rui-Lin Zhao

**Affiliations:** 1 Institue of Ecology, Chinese Research Academy of Environmental Sciences, Beijing 100012, China; 2 State Key Laboratory of Microbial Diversity and Innovative Utilization, Institute of Microbiology, Chinese Academy of Sciences, Beijing 100101, China; 3 Department of Biology, Faculty of Science, Chiang Mai University, Chiang Mai 50200, Thailand; 4 College of Life Sciences, University of Chinese Academy of Sciences, Beijing 101408, China; 5 School of Science, Mae Fah Luang University, Chiang Rai 57100, Thailand; 6 Center of Excellence in Fungal Research, Mae Fah Luang University, Chiang Rai 57100, Thailand; 7 Institute of Microbiology, School of Ecology and Nature Conservation, Beijing Forestry University, Beijing 100083, China

**Keywords:** Beijing, community composition, conservation, macrofungi, species diversity

## Abstract

This study systematically documented macrofungal diversity in Beijing, China (field surveys conducted from 2020 to 2024) using line-transect and random sampling. A total of 1056 species were identified, spanning 2 phyla, 7 classes, 25 orders, 109 families, and 286 genera. The inventory includes 12 new species, 456 new records for Beijing, 79 new records for China, and comprises 116 edible, 56 edible–medicinal, 123 medicinal, and 58 poisonous species. Among these, 542 species were assessed against China’s Macrofungi Redlist, revealing eight species needing conservation attention (seven Near Threatened, one Vulnerable). Analysis revealed stark differences in dominant taxa between natural ecosystems (protected areas) and urban green spaces/parks. In natural areas, macrofungi are dominated by 31 families (e.g., Russulaceae, Cortinariaceae) and 47 genera (e.g., *Russula*, *Cortinarius*). Ectomycorrhizal lineages prevailed, highlighting their critical role in forest nutrient cycling, plant symbiosis, and ecosystem integrity. In urban areas, 10 families (e.g., Agaricaceae, Psathyrellaceae) and 17 genera (e.g., *Leucocoprinus*, *Coprinellus*) were dominant. Saprotrophic genera dominated, indicating their adaptation to decomposing organic matter in human-modified habitats and the provision of ecosystem services. The study demonstrates relatively high macrofungal diversity in Beijing. The distinct functional guild composition—ectomycorrhizal dominance in natural areas versus saprotrophic prevalence in urban zones—reveals complementary ecosystem functions and underscores the conservation value of protected habitats for maintaining vital mycorrhizal networks. These findings provide fundamental data and scientific support for regional biodiversity conservation and sustainable macrofungal resource development.

## 1. Introduction

Fungi are the basis of almost all life on Earth and are widely distributed worldwide. They are essential components of ecosystems and play vital roles in the maintenance of diversity and communities, as well as human development [1,2]. According to the World Fungi Report published by the Royal Botanic Gardens Kew in 2023, it is estimated that the number of fungal species is globally estimated to be between 2 and 3 million, and so far, only 5% to 8% of species have been described or recorded; thus, a large number of fungi are still unknown. Among them, fungi capable of forming large fruiting bodies or sclerotium-like structures with fleshy, membranous, gelatinous, leathery, or woody textures are referred to as macrofungi, which are commonly known as mushrooms [3].

Macrofungi are among the most important lignocellulose-degrading organisms in ecosystems. They not only facilitate nutrient cycling within forest ecosystems but also enhance the uptake of nitrogen, phosphorus, and other essential nutrients by host plants, thereby playing a crucial role in the global carbon cycle [4,5]. Macrofungi can also establish diverse symbiotic relationships with other organisms (such as plants, insects, and algae) and play an indispensable role in maintaining ecological diversity and shaping biological communities [6]. Additionally, macrofungi exhibit medicinal properties, such as antioxidant, antibacterial, and antidiabetic activities [7]. Macrofungi have been one of the most important food sources for human beings since ancient times. Entering a new era, the important concept of the all-encompassing approach to food has been proposed, which involves using plants, animals, and microorganisms for calories and proteins, and developing food resources in an all-around and multi-channel way (https://english.www.gov.cn/, accessed on 17 March 2025). The edible fungus industry has achieved remarkable results from scratch in guaranteeing food security, promoting the development of the modern agricultural industry, and constructing a diversified food supply system. At the same time, with the continuous progress of modern science and technology, the development and utilization of macrofungi in food, medicine, and other aspects have also shown new vitality and have a broad space for development [8,9,10], which put forward higher requirements and challenges for the protection and sustainable utilization of biodiversity in the country and region.

Beijing is located between 115°20′ E–117°32′ E and 39°23′ N–41°05′ N, has an area of approximately 16,410 km^2^, and is characterized by unique natural and geographical conditions [11]. Situated at the northernmost edge of the North China Plain, the city lies at the junction of the Taihang and Yanshan Mountains, forming a semi-enclosed terrain with mountains to the west and north, and open lowlands facing the sea to the east and south [12]. As an ancient city with over 3000 years of urban history and more than 870 years as a capital, Beijing borders Tianjin to the east and is otherwise surrounded by Hebei Province [13,14]. With a permanent population of approximately 21 million, it is a highly internationalized metropolis and one of the most biodiverse major cities in the world [15]. Beijing experiences a warm temperate, semi-humid continental monsoon climate with pronounced seasonal contrasts [16,17]. Summers (June–August) are hot and humid, with mean monthly temperatures often exceeding 26 °C and daily maxima surpassing 35 °C during heat waves [18]. Winters (December–February) are cold and dry, with mean monthly temperatures frequently below 0 °C and extreme lows reaching −15 °C or lower [18,19]. Annual precipitation averages 400–800 mm, over 70% of which occurs during the summer monsoon, resulting in marked seasonal moisture variability [20,21,22,23]. Solar radiation totals approximately 5020–5740 MJ m^−2^ per year, peaking in late spring and early summer but showing a long-term declining trend [18]. Heat resources, measured as accumulated temperature above 0 °C, have increased in recent decades, particularly in the southeastern plains and during spring. Such pronounced seasonal and spatial variations in temperature, precipitation, and radiation strongly shape the ecological suitability of macrofungi across different habitats [18,24,25]. The annual sunshine duration is around 2600 h, with abundant radiation in spring and autumn, frequent cloud cover in summer, and short daylight accompanied by low solar altitude in winter [26]. The soils are diverse, comprising cinnamon soils, fluvo-aquic soils, sandy soils, and anthropogenic fill, and exhibit marked spatial heterogeneity resulting from topography, land use, and human disturbance [11]. They are generally weakly alkaline, with a pH of approximately 8.0 [11]. Urban core areas and roadside greenbelts tend to exhibit higher levels of soil organic carbon (SOC), available phosphorus (AP), and available potassium (AK), whereas some sites affected by traffic and industrial activities show accumulation of lead (Pb) and copper (Cu) [27,28]. Mountain forest soils are rich in organic matter, while soils in shrublands and farmland contain lower but relatively stable nutrient levels [29,30]. Topography strongly influences vegetation patterns, leading to pronounced horizontal and vertical zonation [31]. Mountainous areas are dominated by warm–temperate deciduous broad-leaved forests and coniferous forests, whereas plains and urban zones are primarily covered by artificial green spaces, farmland, and ornamental plantings [32,33,34,35,36]. Dominant species include *Acer truncatum*, *Ginkgo biloba*, *Koelreuteria paniculata*, *Pinus tabuliformis*, and *Robinia pseudoacacia* [37]. The complex topography and diverse river systems have contributed to the rich and unique macrofungal diversity observed in Beijing.

Despite Beijing’s unique natural environment and abundant macrofungal resources, research on macrofungal diversity and resource assessments remains fragmented and largely site-specific or short-term surveys. For example, Liu et al. identified a total of 162 macrofungal species in the Labagoumen nature reserve [38], Zhang et al. conducted a preliminary survey and analysis of macrofungal resources in Badaling Forest Park, Beijing, identifying a total of 127 species [39], and Jin et al. conducted a study on the macrofungi of Xiaoxishan in Beijing by combining field surveys and a literature review, identifying 148 species belonging to 2 phyla, 5 classes, 10 orders, 33 families, and 65 genera [40]. Xu et al. identified 619 species of macrofungi in Beijing [41]. In recent years, many new species and new records of macrofungi have been discovered and reported in Beijing [42,43,44,45,46]. However, wild macrofungal resources have faced an increasing risk of decline due to climate change and human activities. Meanwhile, half of the species have yet to undergo threat status assessments. As an important component of ecosystems, macrofungi play an irreplaceable ecological role in maintaining ecological balance, facilitating nutrient cycling, and indicating environmental health. Biodiversity is closely linked to human well-being; it serves as a vital foundation for human survival and development and represents the lifeblood and cornerstone of Earth’s community of life. Upholding the principle of harmonious coexistence between humans and nature, the Ministry of Ecology and Environment initiated a baseline biodiversity survey in Beijing.

This study, grounded in the natural geographical characteristics of Beijing, was conducted through field investigations from 2020 to 2024. Comprehensive surveys and collections of macrofungi were carried out, and the specimens obtained were identified using DNA barcoding in combination with multi-gene phylogenetic analysis. The objectives of the study were to (1) compile an accurate and comprehensive checklist of macrofungi in Beijing; (2) clarify the species composition of macrofungi in the region; and (3) compare diversity differences between natural ecological environments (protected areas) and urban green spaces and parks. The results provide fundamental data and scientific support for biodiversity conservation, as well as for the sustainable development and utilization of macrofungal resources in Beijing.

## 2. Materials and Methods

### 2.1. Survey Methods

**Figure 1:**
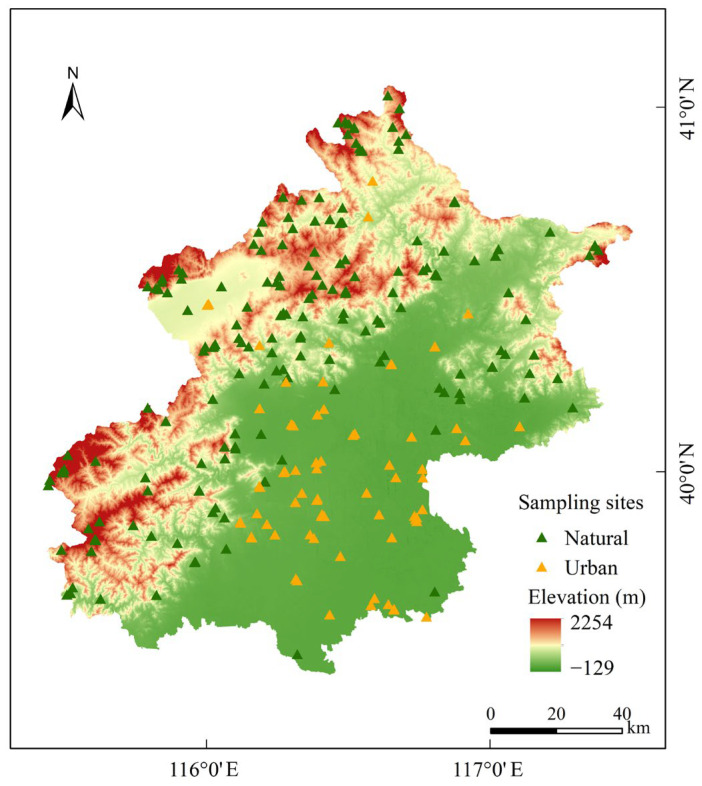
Investigation sites of macrofungi in Beijing.

From 2020 to 2024, macrofungal surveys (such as saprotrophic, mycorrhizal, and parasitic species) were conducted across all 16 districts of Beijing using line-transect and random sampling methods (Figure 1), with green sampling points indicating uninhabited natural environments and orange points representing urban green parks within built-up areas. Survey sites included nature reserves, forest parks, scenic areas, urban green spaces, and city parks. For each specimen collected, metadata such as elevation, geographic coordinates, topography, and ecosystem type were recorded. On the day of collection, specimens were dehydrated in a 50 °C oven for 12 h. After drying, specimens and their corresponding collection numbers were sealed in appropriately sized resealable bags and organized chronologically by collection date. All specimens were deposited in the Herbarium Mycologicum Academiae Sinicae (HMAS) at the Institute of Microbiology, Chinese Academy of Sciences.

### 2.2. Morphological Study

Basidiomata were collected in the field and photographed fresh in their natural habitat. Odor, color changes, macro-morphological features, and chemical reactions of fresh specimens were recorded on site. Microscopic features, including basidiospores, basidia, cheilocystidia, pleurocystidia, and elements of the pileipellis, were examined from dried material of fully mature fruiting bodies. Observations of anatomical and cytological features were conducted using 95% ethanol, 5% KOH, 1% Congo Red, and Melzer’s reagent. Specimens were rehydrated in 5% KOH solution. Spore morphology was observed in Melzer’s reagent. Hymenial cystidia and pileocystidia were stained with 1% aqueous Congo Red [42].

### 2.3. Molecular Phylogenetic Methods

Genomic DNA was extracted from 5 to 20 mg of dried specimen tissue using the Broad-spectrum Plant Rapid Genomic DNA Kit (Biomed, Beijing, China), following the manufacturer’s instructions. Specific primers and target regions were selected based on the characteristics of each specimen for PCR amplification. The amplified products were sequenced by BGI Genomics Co., Ltd. (Beijing, China). The resulting sequences were subjected to BLAST searches on NCBI to obtain preliminary identification results (https://blast.ncbi.nlm.nih.gov/Blast.cgi, accessed on 10 March 2025). For certain taxa, phylogenetic analyses were conducted using reference sequences retrieved from the literature based on the BLAST results, and phylogenetic trees were subsequently constructed [47].

### 2.4. Specimen Identification

Species identification was performed using DNA barcoding in combination with multi-gene phylogenetic analysis. Species names followed Index Fungorum (http://www.indexfungorum.org/, accessed on 11 April 2025), and taxonomic placement was based on the current classification systems of Basidiomycota and Ascomycota [48,49,50].

### 2.5. Species Composition and Resource Evaluation

Based on the species identification results, a statistical analysis of the families and genera of macrofungi in Beijing was conducted. Families containing ten or more species were designated as dominant families, while genera with five or more species were defined as dominant genera [51,52,53]. In combination with a literature review, the economic value and endemism of the recorded macrofungi were also evaluated [54,55]. Visualization of the data was performed using R version 4.4.1. and a Venn diagram showing the overlap of macrofungal species among ecological types [56].

## 3. Results

### 3.1. Species Composition of Macrofungi

Beijing harbors a rich diversity of macrofungal resources. Based on five consecutive years of baseline biodiversity assessments, a comprehensive macrofungal inventory has been established, identifying a total of 1056 species (Table S1), distributed across 2 phyla, 7 classes, 25 orders, 109 families, and 286 genera. Among these, Basidiomycota comprises 3 classes, 18 orders, 95 families, 265 genera, and 1008 species, accounting for 95.45% of the total. Ascomycota includes 4 classes, 7 orders, 14 families, 21 genera, and 48 species, representing 4.55% of the total.

This project has published 12 new species: Candolleomyces luridus, Leucoagaricus bulbosus, Leucoagaricus centrobrunneolus, Leucoagaricus cinereibisporus, Leucoagaricus luteocanus, Leucoagaricus subcandidus, Leucoagaricus subnivalis, Leucoagaricus testaceumbonatus, Leucoagaricus xantholepis, Leucocoprinus beijingensis, Leucocoprinus digitatocystis, and Russula paragraveolens [42,47,57].

A total of 456 species were identified as new records in Beijing, primarily including 36 species of *Inocybe*, 23 of *Entoloma*, 20 of *Russula*, 16 of *Cortinarius*, 13 of *Agaricus*, 13 of *Amanita*, 13 of *Marasmius*, 11 of *Hebeloma*, 11 of *Leucocoprinus*, and 10 of *Helvella*, among others. The checklist of new records in Beijing is given below. Author abbreviations of scientific names follow the format of the Authors of Fungal Names (https://nmdc.cn/fungalnames/, accessed on 23 June 2025).

1. *Agaricus aridicola* Geml, Geiser & Royse ex Mateos, J. Morales, J.A. Muñoz, Rey & C. Tovar NE
2. *Agaricus deardorffensis* Kerrigan NE
3. *Agaricus jacobi* L.A. Parra, A. Caball. & Callac NE
4. *Agaricus latiumbonatus* S. Hussain NE
5. *Agaricus litoralis* (Wakef. & A. Pearson) Pilát LC E
6. *Agaricus luteopileus* R.L. Zhao & B. Cao NE
7. *Agaricus macrocarpus* F.H. Møller DD
8. *Agaricus malangelus* Kerrigan NE
9. *Agaricus menieri* Bon NE
10. *Agaricus microviolaceus* M.Q. He & R.L. Zhao NE
11. *Agaricus pseudopratensis* (Bohus) Wasser DD
12. *Agaricus tephrolepidus* L.A. Parra, C. Billette, Angelini, G. Mata & Callac NE
13. *Agaricus thiersii* Kerrigan & Vellinga NE
14. *Agrocybe arvalis* (Fr.) Singer LC
15. *Agrocybe rivulosa* Nauta NE
16. *Agrocybe vervacti* (Fr.) Singer DD
17. *Aleuria aurantia* (Pers.) Fuckel LC
18. *Aleurodiscus dextrinoideocerussatus* Manjón, M.N. Blanco & G. Moreno NE
19. *Amanita aurora* Härkönen & Niemelä NE
20. *Amanita caesareoides* Lj.N. Vassiljeva LC E
21. *Amanita ceciliae* (Berk. & Broome) Bas LC
22. *Amanita fense* M. Mu & L.P. Tang NE
23. *Amanita fulvoides* Neville & Poumarat NE
24. *Amanita incarnatifolia* Zhu L. Yang DD P
25. *Amanita magniverrucata* Thiers & Ammirati NE
26. *Amanita manginiana* Har. & Pat. LC P
27. *Amanita morrisii* Peck NE
28. *Amanita oberwinkleriana* Zhu L. Yang & Yoshim. Doi LC P
29. *Amanita ovalispora* Boedijn LC
30. *Amanita phalloides* (Vaill. ex Fr.) Link DD
31. *Amanita rubescens* Pers. LC
32. *Aseroe coccinea* Imazeki & Yoshimi ex Kasuya DD
33. *Aureoboletus zangii* X.F. Shi & P.G. Liu DD
34. *Boletus carminiporus* Bessette, Both & Dunaway NE
35. *Boletus subfraternus* Coker & Beers NE
36. *Bonomyces arnoldii* (Boud.) P.-A. Moreau, Vizzini & P. Alvarado NE
37. *Bovista capensis* (Fr.) J.C. Coetzee & A.E. van Wyk NE
38. *Bovista nigrescens* Pers. LC M
39. *Butyriboletus peckii* (Frost) Kuan Zhao & Zhu L. Yang DD
40. *Butyriboletus subregius* Yang Wang, Bo Zhang & Yu Li NE
41. *Calocybe aurantiaca* X.D. Yu & Jia J. Li NE
42. *Calocybe convexa* X.D. Yu & Jia J. Li NE
43. *Calocybe cyanella* Singer ex Redhead & Singer NE
44. *Calocybe fulvipes* J.Z. Xu & Yu Li NE
45. *Calocybe hebelomoides* (Ew. Gerhardt) Læssøe & J.H. Petersen NE
46. *Calocybe indica* Purkay. & A. Chandra NE
47. *Calocybe obscurissima* (A. Pearson) M.M. Moser NE
48. *Calocybe ochracea* (R. Haller Aar.) Bon DD
49. *Calvatia subbooniana* R.L. Zhao & J.Xin Li NE
50. *Candolleomyces aberdarensis* (A. Melzer, Kimani & R. Ullrich) D. Wächt. & A. Melzer NE
51. *Candolleomyces cacao* (Desjardin & B.A. Perry) D. Wächt. & A. Melzer NE
52. *Candolleomyces subcandolleanus* C.L. Hou & Hao Zhou NE
53. *Candolleomyces sulcatotuberculosus* (J. Favre) D. Wächt. & A. Melzer NE
54. *Cantharellus appalachiensis* R.H. Petersen DD
55. *Cantharellus roseocanus* (Redhead, Norvell & Danell) Redhead, Norvell & Moncalvo NE
56. *Chamaemyces fracidus* (Fr.) Donk DD
57. *Clavaria flavipes* Pers. LC
58. *Clavulinopsis laeticolor* (Berk. & M.A. Curtis) R.H. Petersen DD
59. *Clitocybe lamoureae* Armada, Bellanger, Bidaud & P.-A. Moreau NE
60. *Clitocybe lohjaensis* Harmaja NE
61. *Clitopilus cretoalbus* A. Izhar, Zaman, M. Asif, H. Bashir, Niazi & Khalid NE
62. *Clitopilus geminus* (Paulet) Noordel. & Co-David DD
63. *Clitopilus piperatus* (G. Stev.) Noordel. & Co-David NE
64. *Collybia cirrhata* (Schumach.) Quél. DD
65. *Collybia cookei* (Bres.) J.D. Arnold LC
66. *Collybiopsis dichroa* (Berk. & M.A. Curtis) R.H. Petersen NE
67. *Collybiopsis gibbosa* (Corner) R.H. Petersen NE
68. *Collybiopsis indocta* (Corner) R.H. Petersen NE
69. *Collybiopsis nonnulla* (Corner) R.H. Petersen NE
70. *Coltricia weii* Y.C. Dai DD
71. *Coniophora hanoiensis* Pat. NE
72. *Conobolbitina dasypus* (Romagn.) T. Bau & H.B. Song NE
73. *Conocybe brachypodii* (Velen.) Hauskn. & Svrček NE
74. *Conocybe deliquescens* Hauskn. & Krisai NE
75. *Conocybe fuscimarginata* (Murrill) Singer NE
76. *Conocybe mesospora* Kühner ex Watling DD
77. *Conocybe microspora* (Velen.) Dennis NE
78. *Conocybe praticola* E.F. Malysheva NE
79. *Conocybe semiglobata* Kühner & Watling DD
80. *Conocybe subcrispa* (Murrill) Singer NE
81. *Conocybe vestita* (Fr.) Kühner NE
82. *Coprinellus ovatus* M. Kamran & S. Jabeen NE
83. *Coprinopsis bellula* (Uljé) P. Roux & Eyssart. NE
84. *Coprinopsis friesii* (Quél.) P. Karst. LC M
85. *Coprinopsis fusispora* L. Nagy, Vágvölgyi & Papp NE
86. *Coprinopsis lagopus* (Fr.) Redhead, Vilgalys & Moncalvo LC M
87. *Coprinopsis sclerotiorum* (Horvers & de Cock) Redhead, Vilgalys & Moncalvo NE
88. *Coprinopsis stangliana* (Enderle, Bender & Gröger) Redhead, Vilgalys & Moncalvo NE
89. *Coprinopsis strossmayeri* (Schulzer) Redhead, Vilgalys & Moncalvo NE
90. *Coprinopsis udicola* Örstadius, A. Melzer & E. Larss. NE
91. *Coprinus fissolanatus* R.F.O. Kemp NE
92. *Cordyceps tenuipes* (Peck) Kepler, B. Shrestha & Spatafora NE E
93. *Cortinarius acutus* (Pers.) Fr. NE
94. *Cortinarius alboviolaceus* (Pers.) Fr. DD E
95. *Cortinarius confirmatus* Rob. Henry NE
96. *Cortinarius decipiens* (Pers.) Fr. DD
97. *Cortinarius disjungendus* P. Karst. NE
98. *Cortinarius epipurrus* Chevassut & Rob. Henry NE
99. *Cortinarius fulvopaludosus* Kytöv., Niskanen & Liimat. NE
100. *Cortinarius gallurae* D. Antonini, M. Antonini & Consiglio NE
101. *Cortinarius melanotus* Kalchbr. DD
102. *Cortinarius murinascens* Kytöv., Niskanen & Liimat. NE
103. *Cortinarius persoonianus* Bidaud NE
104. *Cortinarius subferrugineus* (Batsch) Fr. DD
105. *Cortinarius trivialis* J.E. Lange LC
106. *Cortinarius umbrinolutescens* Reumaux NE
107. *Cortinarius uraceomajalis* Dima, Liimat., Niskanen & Bojantchev NE
108. *Cortinarius violaceus* (L.) Gray LC
109. *Crepidotus indicus* A.M. Kumar & C.K. Pradeep NE
110. *Crustomyces expallens* (Bres.) Hjortstam DD
111. *Cuphophyllus borealis* (Peck) Bon ex Courtec. NE
112. *Cyanoboletus pulverulentus* (Opat.) Gelardi, Vizzini & Simonini LC
113. *Cyathus apiculatus* M.M.B. Barbosa & Baseia NE
114. *Cyathus bulleri* H.J. Brodie NE
115. *Cyathus jiayuguanensis* J. Yu, T.X. Zhou & L.Z. Zhao DD
116. *Cyclocybe erebioides* Angelini & Vizzini NE
117. *Cystolepiota eriophora* (Peck) Knudsen DD
118. *Deconica coprophila* (Bull.) P. Karst. LC
119. *Deconica velifera* (J. Favre) Noordel. NE
120. *Dendrothele incrustans* (P.A. Lemke) P.A. Lemke NE
121. *Descolea quercina* J. Khan & Naseer NE
122. *Echinoderma jacobi* (Vellinga & Knudsen) Gminder DD
123. *Entocybe nitida* (Quél.) T.J. Baroni, Largent & V. Hofst. LC
124. *Entoloma aprile* (Britzelm.) Sacc. DD
125. *Entoloma aurorae-borealis* Noordel., Weholt, Eidissen & Lorås NE
126. *Entoloma bicolor* Murrill NE
127. *Entoloma brunneorugulosum* Reschke, Noordel. & Lotz-Winter NE
128. *Entoloma bryorum* Romagn. NE
129. *Entoloma caccabus* (Kühner) Noordel. DD
130. *Entoloma cyaneolilacinum* Noordel., J.B. Jordal, Brandrud & Dima NE
131. *Entoloma formosum* (Fr.) Noordel. DD
132. *Entoloma fuscosquamosum* Hesler NE
133. *Entoloma griseopruinatum* Noordel. & Cheype NE
134. *Entoloma griseorugulosum* Noordel. & Fern. Sas. NE
135. *Entoloma incanum* (Fr.) Hesler LC P
136. *Entoloma incarnatofuscescens* (Britzelm.) Noordel. NE
137. *Entoloma indutoides* (P.D. Orton) Noordel. NE
138. *Entoloma mediterraneense* Noordel. & Hauskn. DD
139. *Entoloma neglectum* (Lasch) Arnolds DD
140. *Entoloma nigrovelutinum* O.V. Morozova & A.V. Alexandrova NE
141. *Entoloma omiense* (Hongo) E. Horak LC P
142. *Entoloma rivulare* Kokkonen NE
143. *Entoloma rusticoides* (Gillet) Noordel. DD
144. *Entoloma subaraneosum* Xiao L. He & T.H. Li DD
145. *Entoloma subradiatum* (Kühner & Romagn.) M.M. Moser DD
146. *Entoloma undulatosporum* Arnolds & Noordel. NE
147. *Fasciodontia brasiliensis* Yurchenko & Riebesehl NE
148. *Fibrodontia gossypina* Parmasto NE
149. *Floccularia albolanaripes* (G.F. Atk.) Redhead LC E
150. *Fomitiporia carpinea* X.H. Ji, X.M. Tian & Y.C. Dai NE
151. *Fomitopsis marianiae* (Bres.) Spirin, Vlasák & Cartabia NE
152. *Fomitopsis monomitica* (Yuan Y. Chen) Spirin & Viner NE
153. *Fomitopsis officinalis* (Vill.) Bondartsev & Singer DD M
154. *Geastrum argentinum* Speg. NE
155. *Geastrum floriforme* Vittad. LC
156. *Geastrum fuscoglebum* (Zeller) Jeppson & E. Larss. NE
157. *Geastrum gorgonicum* M.P. Martín, M. Dueñas & Telleria NE
158. *Geastrum minimum* Schwein. LC
159. *Geastrum pectinatum* Pers. LC
160. *Geastrum smardae* V.J. Staněk NE
161. *Geastrum velutinum* Morgan LC M
162. *Geopora cervina* (Velen.) T. Schumach. NE
163. *Gerronema confusum* L. Fan & T.Y. Zhao NE
164. *Gerronema kuruvense* K.P.D. Latha & Manim. NE
165. *Gymnopilus flavus* (Bres.) Singer DD
166. *Gymnopilus sapineus* (Fr.) Murrill LC
167. *Gymnopus brunneodiscus* Antonín, Ryoo & Ka NE
168. *Gymnopus cremeostipitatus* Antonín, Ryoo & Ka NE
169. *Gymnopus foetidus* (Sowerby) P.M. Kirk LC
170. *Gymnopus montagnei* (Berk.) Redhead NE
171. *Gymnopus sepiiconicus* (Corner) A.W. Wilson, Desjardin & E. Horak NE
172. *Gymnopus subsulphureus* (Peck) Murrill NE
173. *Gyroporus austrobrasiliensis* A.C. Magnago & R.M. Silveira NE
174. *Gyroporus subglobosus* N.K. Zeng, H.J. Xie, L.P. Tang & M. Mu NE
175. *Hebeloma affine* A.H. Sm., V.S. Evenson & Mitchel NE
176. *Hebeloma album* Peck DD
177. *Hebeloma ammophilum* Bohus NE
178. *Hebeloma asperosporum* Beker & U. Eberh. NE
179. *Hebeloma eburneum* Malençon NE
180. *Hebeloma flaccidum* A.H. Sm., V.S. Evenson & Mitchel NE
181. *Hebeloma hiemale* Bres. LC
182. *Hebeloma neurophyllum* G.F. Atk. NE
183. *Hebeloma pseudofragilipes* Beker, Vesterh. & U. Eberh. NE
184. *Hebeloma sordescens* Vesterh. NE
185. *Hebeloma testaceum* Batsch ex Quél. DD
186. *Helvella alborava* L. Fan, N. Mao & H. Zhou NE
187. *Helvella bicolor* Raddi NE
188. *Helvella calycina* Skrede, T.A. Carlsen & T. Schumach. NE
189. *Helvella compressa* (Snyder) N.S. Weber NE
190. *Helvella danica* Skrede, T.A. Carlsen & T. Schumach. NE
191. *Helvella fibrosa* (Wallr.) Korf NE
192. *Helvella floriforma* Q. Zhao & K.D. Hyde NE
193. *Helvella liui* X.C. Wang & W.Y. Zhuang NE
194. *Helvella pseudopezizoides* L. Fan, N. Mao & Y.Y. Xu NE
195. *Helvella sublicia* Holmsk. NE
196. *Hemimycena cucullata* (Pers.) Singer DD
197. *Hemimycena mairei* (E.-J. Gilbert) Singer NE
198. *Hohenbuehelia atrocoerulea* (Fr.) Singer DD
199. *Hortiboletus amygdalinus* Xue T. Zhu & Zhu L. Yang NE
200. *Hortiboletus rufosquamosus* L. Fan, N. Mao & T.Y. Zhao NE
201. *Hydnum vesterholtii* Olariaga, Grebenc, Salcedo & M.P. Martín NE
202. *Hygrocybe acutoconica* (Clem.) Singer LC
203. *Hygrocybe coccineocrenata* (P.D. Orton) M.M. Moser DD
204. *Hygrocybe singeri* (A.H. Sm. & Hesler) Singer NE
205. *Hymenogaster arenarius* Tul. & C. Tul. DD
206. *Hymenopellis biyangensis* Y.J. Liu, B. Zhang & Xiao Li NE
207. *Hymenopellis rubrobrunnescens* (Redhead, Ginns & Shoemaker) R.H. Petersen NE
208. *Hymenopellis utriformis* Niego & Raspé NE
209. *Hyphodontia tropica* Sheng H. Wu NE
210. *Hypholoma acutum* (Sacc.) E. Horak DD
211. *Hypholoma fasciculare* (Huds.) P. Kumm. LC M, P
212. *Hypomyces armeniacus* Tul. NE
213. *Hypomyces chlorinus* Tul. NE
214. *Hypoxylon guilanense* Pourmogh. & C. Lamb. NE
215. *Hypoxylon rubiginosum* (Pers.) Fr. LC
216. *Infundibulicybe trachyspora* J.Z. Xu, J.C. Qin & Yu Li NE
217. *Inocybe aeruginascens* Babos NE
218. *Inocybe amblyospora* Kühner NE
219. *Inocybe amelandica* Bandini & B. Oertel NE
220. *Inocybe amethystina* Kuyper DD
221. *Inocybe armeniaca* Huijsman NE
222. *Inocybe assimilata* Britzelm. LC
223. *Inocybe bellidiana* Bandini, B. Oertel & U. Eberh. NE
224. *Inocybe bombina* Bandini & B. Oertel NE
225. *Inocybe brunnea* Quél. DD P
226. *Inocybe calospora* Quél. LC
227. *Inocybe caroticolor* T. Bau & Y.G. Fan DD
228. *Inocybe castanea* Peck NE
229. *Inocybe castaneicolor* A. La Rosa, Bizio, Saitta & Tedersoo NE
230. *Inocybe curvipes* P. Karst. LC
231. *Inocybe decemgibbosa* (Kühner) Vauras NE
232. *Inocybe dryadiana* Bandini & B. Oertel NE
233. *Inocybe fibrosoides* Kühner NE
234. *Inocybe grusiana* Bandini & B. Oertel NE
235. *Inocybe lampetiana* Bandini & B. Oertel NE
236. *Inocybe latibulosa* E. Horak, Matheny & Desjardin NE
237. *Inocybe leptocystis* G.F. Atk. LC
238. *Inocybe minutissima* Carteret & Reumaux NE
239. *Inocybe morganae* Bandini, B. Oertel & U. Eberh. NE
240. *Inocybe oblectabilis* (Britzelm.) Sacc. NE
241. *Inocybe obscurobadia* (J. Favre) Grund & D.E. Stuntz DD
242. *Inocybe perlucida* Bandini & E. Ferrari NE
243. *Inocybe plocamophora* Singer, I.J.A. Aguiar & Ivory NE
244. *Inocybe pruinosa* R. Heim DD
245. *Inocybe pseudoreducta* Stangl & Glowinski NE
246. *Inocybe putilla* Bres. DD
247. *Inocybe sambucella* G.F. Atk. NE
248. *Inocybe serotina* Peck NE
249. *Inocybe sindonia* (Fr.) P. Karst. DD
250. *Inocybe stellata* E. Horak, Matheny & Desjardin NE
251. *Inocybe suryana* Bandini & B. Oertel NE
252. *Inocybe terrifera* Kühner NE
253. *Laccaria fulvogrisea* Popa, Rexer & G. Kost DD E
254. *Laccaria ochropurpurea* (Berk.) Peck DD
255. *Lacrymaria glareosa* (J. Favre) Watling NE
256. *Lacrymaria hypertropicalis* (Guzmán, Bandala & Montoya) Cortez NE
257. *Lacrymaria pyrotricha* (Holmsk.) Konrad & Maubl. DD
258. *Lactarius aspideus* (Fr.) Fr. DD
259. *Lactarius evosmus* Kühner & Romagn. NE
260. *Lactarius hatsudake* Nobuj. Tanaka LC E, M
261. *Lactarius mediterraneensis* Llistos. & Bellù NE
262. *Lactarius microbuccinatus* H. Lee, Wisitr. & Y.W. Lim NE
263. *Lactarius mitratus* H. Lee, Wisitr. & Y.W. Lim NE
264. *Lactarius quietus* (Fr.) Fr. LC E, M
265. *Lactarius rubidus* (Hesler & A.H. Sm.) Methven NE
266. *Lactarius scrobiculatus* (Scop.) Fr. LC P
267. *Lepiota andegavensis* Mornand NE
268. *Lepiota atrobrunneodisca* L. Fan, L. Xia & N. Mao NE
269. *Lepiota atrodisca* Zeller DD
270. *Lepiota castaneidisca* Murrill NE
271. *Lepiota forquignonii* Quél. NE
272. *Lepiota oreadiformis* Velen. DD
273. *Lepiota pallidiochracea* J.F. Liang & Zhu L. Yang DD
274. *Lepiota psalion* Huijser & Vellinga NE
275. *Lepiota xanthophylla* P.D. Orton DD
276. *Lepista panaeola* (Fr.) P. Karst. NE
277. *Lepista panaeolus* (Fr.) P. Karst. DD
278. *Lepista ricekii* Bon DD
279. *Leucoagaricus japonicus* (Kawam. ex Hongo) Hongo NE
280. *Leucoagaricus purpurascens* T. Guo & Z.W. Ge NE
281. *Leucoagaricus roseilividus* (Murrill) E. Ludw. NE
282. *Leucocoprinus albosquamosus* (Y.R. Ma, Z.W. Ge & T.Z. Liu) M. Asif, Saba & Vellinga NE
283. *Leucocoprinus amanitoides* (R.M. Davis & Vellinga) Kun L. Yang, Jia Ying Lin & Zhu L. Yang NE
284. *Leucocoprinus atroazureus* (J.F. Liang, Zhu L. Yang & J. Xu) M. Asif, Saba & Vellinga NE
285. *Leucocoprinus barssii* (Zeller) Migl. & Donato NE
286. *Leucocoprinus caeruleovertens* (Justo, Bizzi & Angelini) M. Asif, Saba & Vellinga NE
287. *Leucocoprinus cretaceus* (Bull.) Locq. DD
288. *Leucocoprinus lahorensiformis* (S. Hussain, H. Ahmad, Afshan & Khalid) M. Asif, Saba & Vellinga NE
289. *Leucocoprinus pakistaniensis* (S. Jabeen & A.N. Khalid) M. Asif & Saba NE
290. *Leucocoprinus rubrobrunneus* (E.F. Malysheva, Svetash. & Bulakh) M. Asif, Saba & Vellinga NE
291. *Leucocoprinus subvolvatus* (Malençon & Bertault) M. Asif, Saba & Vellinga NE
292. *Leucocoprinus viriditinctus* (Berk. & Broome) M. Asif, Saba & Vellinga NE
293. *Litschauerella gladiola* (G. Cunn.) Stalpers & P.K. Buchanan DD
294. *Lycoperdon curtisii* Berk. NE
295. *Lycoperdon glabrescens* Berk. DD E
296. *Lycoperdon subincarnatum* Peck DD E
297. *Macrocybe gigantea* (Massee) Pegler & Lodge NT E, M
298. *Macrolepiota orientiexcoriata* Z.W. Ge, Zhu L. Yang & Vellinga DD
299. *Macropsalliota americana* (Peck) Kun L. Yang, Jia Ying Lin & Zhu L. Yang NE
300. *Mallocybe fulvoumbonata* (Murrill) Matheny & Esteve-Rav. NE
301. *Mallocybe leucoblema* (Kühner) Matheny & Esteve-Rav. NE
302. *Mallocybe leucoloma* (Kühner) Matheny & Esteve-Rav. NE
303. *Mallocybe leucothrix* Matheny & M.E. Sm. NE
304. *Mallocybe myriadophylla* (Vauras & E. Larss.) Matheny & Esteve-Rav. NE
305. *Mallocybe siciliana* (Brugaletta, Consiglio & M. Marchetti) Brugaletta, Consiglio & M. Marchetti NE
306. *Mallocybe subtomentosa* (Peck) Matheny & Kudzma NE
307. *Mallocybe unicolor* (Peck) Matheny & Esteve-Rav. NE
308. *Marasmiellus brunneigracilis* (Corner) J.S. Oliveira NE
309. *Marasmiellus rhizomorphogenus* Antonín, Ryoo & H.D. Shin NE
310. *Marasmius albopurpureus* T.H. Li & C.Q. Wang LC
311. *Marasmius brunneospermus* Har. Takah. NE
312. *Marasmius collinus* (Scop.) Singer DD
313. *Marasmius crinipes* Antonín, Ryoo & H.D. Shin NE
314. *Marasmius delectans* Morgan NE
315. *Marasmius fulvoferrugineus* Gilliam NE
316. *Marasmius laticlavatus* Wannathes, Desjardin & Lumyong NE
317. *Marasmius occultatiformis* Antonín, Ryoo & H.D. Shin DD
318. *Marasmius occultatus* Har. Takah. NE
319. *Marasmius ochroleucus* Desjardin & E. Horak DD
320. *Marasmius pallescens* Murrill NE
321. *Marasmius strobiluriformis* Antonín, Ryoo & H.D. Shin NE
322. *Marasmius tangerinus* Wannathes, Suwannar., Kumla & Lumyong NE
323. *Melanoleuca griseobrunnea* Antonín, Ďuriška & Tomšovský NE
324. *Melanoleuca juliannae* Rimóczi, Antonín, L. Nagy & Tomšovský NE
325. *Melanoleuca microcephala* (P. Karst.) Singer NE
326. *Melanophyllum haematospermum* (Bull.) Kreisel DD
327. *Mycena cicognanii* Robich NE
328. *Mycena megaspora* Kauffman NE
329. *Mycena niveipes* (Murrill) Murrill DD
330. *Mycena pearsoniana* Dennis ex Singer NE
331. *Mycena plumbea* P. Karst. NE
332. *Mycena xantholeuca* Kühner NE
333. *Mycenella bryophila* (Voglino) Singer DD
334. *Mycetinis scorodonius* (Fr.) A.W. Wilson & Desjardin LC E, M
335. *Neoboletus erythropus* (Pers.) C. Hahn NE
336. *Panaeolus acuminatus* (P. Kumm.) Quél. LC P
337. *Panaeolus olivaceus* F.H. Møller NE
338. *Panus purpuratus* G. Stev. NE
339. *Paragalactinia succosa* (Berk.) Van Vooren NE
340. *Paralepista gilva* (Pers.) Raithelh. DD
341. *Paramarasmius palmivorus* (Sharples) Antonín & Kolařík NE
342. *Parasola auricoma* (Pat.) Redhead, Vilgalys & Hopple DD
343. *Parasola conopilea* (Fr.) Örstadius & E. Larss. NE
344. *Parasola conopilus* (Fr.) Örstadius & E. Larss. DD
345. *Parasola kuehneri* (Uljé & Bas) Redhead, Vilgalys & Hopple NE
346. *Parasola lactea* (A.H. Sm.) Redhead, Vilgalys & Hopple NE
347. *Parasola lilatincta* (Bender & Uljé) Redhead, Vilgalys & Hopple NE
348. *Parasola malakandensis* S. Hussain, Afshan & H. Ahmad NE
349. *Parasola psathyrelloides* K.G.G. Ganga & Manim. NE
350. *Parasola setulosa* (Berk. & Broome) Redhead, Vilgalys & Hopple NE
351. *Paxillus vernalis* Watling NE
352. *Peniophora bicornis* Hjortstam & Ryvarden DD
353. *Peniophora incarnata* (Pers.) P. Karst. LC
354. *Peniophora lassa* (Spirin & Kout) Y.Lin Xu & S.H. He NE
355. *Perenniporia corticola* (Corner) Decock NE
356. *Perenniporia formosana* T.T. Chang DD
357. *Peziza depressa* Pers. DD
358. *Peziza irina* Quél. NE
359. *Phaeoclavulina decurrens* (Pers.) J.H. Petersen NE
360. *Phallus hadriani* Vent. DD
361. *Phallus ultraduplicatus* X.D. Yu, W. Lv, S.X. Lv, Xu H. Chen & Qin Wang NE
362. *Phanerochaete sanguineocarnosa* Floudas & Hibbett NE
363. *Phellinus erectus* A. David, Dequatre & Fiasson DD
364. *Phloeomana minutula* (Sacc.) Redhead LC
365. *Pholiota limonella* (Peck) Sacc. DD
366. *Pholiotina calongei* Siquier, E. Suárez, Salom & Planas NE
367. *Pluteus castroae* Justo & E.F. Malysheva NE
368. *Pluteus decoloratus* E. Horak NE
369. *Pluteus fibrillosus* Murrill NE
370. *Pluteus inflatus* Velen. NE
371. *Pluteus multiformis* Justo, A. Caball. & G. Muñoz NE
372. *Pluteus pantherinus* Courtec. & M. Uchida DD
373. *Pluteus romellii* (Britzelm.) Sacc. DD
374. *Pluteus siccus* E.F. Malysheva NE
375. *Polyporus gracilisporus* (H. Lee, N.K. Kim & Y.W. Lim) Bernicchia & Gorjón NE
376. *Polyporus mori* (Pollini) Fr. NE M
377. *Protoglossum niveum* (Vittad.) T.W. May DD
378. *Psathyrella atricastanea* (Murrill) A.H. Sm. NE
379. *Psathyrella pygmaea* (Bull.) Singer DD
380. *Psathyrella thiersii* A.H. Sm. DD
381. *Psathyrella uliginicola* McKnight & A.H. Sm. NE
382. *Psathyrella vestita* (Peck) A.H. Sm. NE
383. *Pseudosperma amabile* Bandini, B. Oertel & Wehr NE
384. *Pseudosperma citrinostipes* Y.G. Fan & W.J. Yu NE
385. *Pseudosperma melliolens* (Kühner) Matheny & Esteve-Rav. NE
386. *Pseudosperma perlatum* (Cooke) Matheny & Esteve-Rav. NE
387. *Pseudosperma solare* Bandini, B. Oertel & U. Eberh. NE
388. *Pseudosperma sororium* (Kauffman) Matheny & Esteve-Rav. NE
389. *Ptychogaster rubescens* Boud. NE
390. *Ramaria paraconcolor* Franchi & M. Marchetti NE
391. *Ramaria pseudogracilis* R.H. Petersen NE
392. *Ramaria stricta* (Pers.) Quél. NT E
393. *Rhodocollybia maculata* (Alb. & Schwein.) Singer LC E
394. *Rhodocybe asyae* Seslı & Vizzini NE
395. *Rhodocybe formosa* Vila, Contu, F. Caball. & A. Ortega NE
396. *Rhodocybe griseonigrella* (Vila, Contu, F. Caball. & Ribes) Vizzini, Vila, Picillo & Contu NE
397. *Russula albonigra* (Krombh.) Fr. LC E, M
398. *Russula chloroides* (Krombh.) Bres. LC E
399. *Russula cremicolor* G.J. Li & C.Y. Deng NE
400. *Russula emeticicolor* Jul. Schäff. DD
401. *Russula faustiana* Sarnari NE
402. *Russula fluvialis* Taipale, Ruots. & Kälviäinen NE
403. *Russula foetentula* Peck NE
404. *Russula laricina* Velen. DD
405. *Russula livescens* (Batsch) Bataille LC E
406. *Russula mustelina* Fr. LC E
407. *Russula nitida* (Pers.) Fr. LC E
408. *Russula pelargonia* Niolle DD
409. *Russula postiana* Romell NE
410. *Russula pseudoamoenicolor* A. Ghosh, Buyck, K. Das, Baghela & R.P. Bhatt NE
411. *Russula puellaris* Fr. LC E
412. *Russula quercicola* Razzaq, Shahid, Naseer & Khalid NE
413. *Russula solaris* Ferd. & Winge LC
414. *Russula variicolor* Murrill NE
415. *Russula vesca* Fr. LC E, M
416. *Russula violacea* Quél. LC E
417. *Sarcoscypha javensis* Höhn. DD
418. *Scleroderma meridionale* Demoulin & Malençon NE
419. *Scleroderma nastii* Raut NE
420. *Scleroderma septentrionale* Jeppson NE
421. *Scutellinia scutellata* (L.) Lambotte LC
422. *Scytinostroma portentosum* (Berk. & M.A. Curtis) Donk DD
423. *Singerocybe adirondackensis* (Peck) Zhu L. Yang & J. Qin NE
424. *Sinoperdon caudatum* (J. Schröt.) R.L. Zhao & J.Xin Li NE
425. *Sistotrema confluens* Pers. DD
426. *Skeletocutis ochroalba* Niemelä DD
427. *Spodocybe trulliformis* (Fr.) Vizzini, P. Alvarado & Dima NE
428. *Spongipellis variispora* Spirin, Miettinen & Vlasák NE
429. *Steccherinum hirsutum* Y.X. Wu & C.L. Zhao NE
430. *Stereum complicatum* (Fr.) Fr. NE
431. *Stropharia hardii* G.F. Atk. DD
432. *Suillus collinitus* (Fr.) Kuntze DD E, M
433. *Tarzetta sepultarioides* Van Vooren NE
434. *Tatraea macrospora* (Peck) Baral NE
435. *Tricholoma equestre* (L.) P. Kumm. LC
436. *Tricholoma frondosae* Kalamees & Shchukin NE
437. *Tricholosporum haitangshanum* Yu Li & J.Z. Xu NE
438. *Tubaria romagnesiana* Arnolds NE
439. *Tulosesus hiascens* (Fr.) D. Wächt. & A. Melzer NE
440. *Tulostoma fimbriatum* Fr. LC
441. *Tulostoma fulvellum* Bres. NE
442. *Tulostoma squamosum* (J.F. Gmel.) Pers. NE
443. *Tylopilus himalayanus* D. Chakr., K. Das & Vizzini NE
444. *Utraria decipiens* (Durieu & Mont.) R.L. Zhao & J.Xin Li NE
445. *Utraria lambinonii* (Demoulin) R.L. Zhao & J.Xin Li NE
446. *Utraria nivea* (Kreisel) R.L. Zhao & J.Xin Li NE
447. *Utraria pulcherrima* (Berk. & M.A. Curtis) R.L. Zhao & J.Xin Li NE
448. *Volvariella bombycina* (Schaeff.) Singer LC E, M
449. *Volvariella clavocystidiata* Kapitonov & E.F. Malysheva NE
450. *Volvariella hypopithys* (Fr.) Shaffer DD
451. *Volvariella murinella* (Quél.) M.M. Moser ex Dennis, P.D. Orton & Hora DD
452. *Volvariella subtaylori* Hongo NE
453. *Volvariella terrea* Musumeci & A. Riva NE
454. *Volvopluteus earlei* (Murrill) Vizzini, Contu & Justo NE
455. *Xanthoperenniporia tenuis* (Schwein.) B.K. Cui & Xing Ji NE
456. *Xerocomellus pruinatus* (Fr. & Hök) Šutara NE

Notes: DD: Data deficient; LC: Least concern; NE: Not evaluated; NT: Near threatened; E: Edible; M: Medicinal; P: Poisonous.

A total of 79 new records for China were discovered, primarily including 13 species of *Inocybe*, 8 of *Entoloma*, 5 of *Pluteus*, 4 of *Hebeloma*, 3 of *Amanita*, 3 of *Leucocoprinus*, and 3 of *Marasmius*, among others. The checklist of new records in China is given below.

1. Agaricus latiumbonatus S. Hussain
2. Agaricus menieri Bon
3. Amanita aurora Härkönen & Niemelä
4. Amanita magniverrucata Thiers & Ammirati
5. Amanita morrisii Peck
6. Boletus subfraternus Coker & Beers
7. Clitocybe lamoureae Armada, Bellanger, Bidaud & P.-A. Moreau
8. Clitocybe lohjaensis Harmaja
9. Clitopilus cretoalbus A. Izhar, Zaman, M. Asif, H. Bashir, Niazi & Khalid
10. Conocybe vestita (Fr.) Kühner
11. Coprinopsis sclerotiorum (Horvers & de Cock) Redhead, Vilgalys & Moncalvo
12. Coprinopsis stangliana (Enderle, Bender & Gröger) Redhead, Vilgalys & Moncalvo
13. Cortinarius fulvopaludosus Kytöv., Niskanen & Liimat.
14. Cortinarius persoonianus Bidaud
15. Cyathus apiculatus M.M.B. Barbosa & Baseia
16. Dendrothele incrustans (P.A. Lemke) P.A. Lemke
17. Entoloma aurorae-borealis Noordel., Weholt, Eidissen & Lorås
18. Entoloma brunneorugulosum Reschke, Noordel. & Lotz-Winter
19. Entoloma bryorum Romagn.
20. Entoloma cyaneolilacinum Noordel., J.B. Jordal, Brandrud & Dima
21. Entoloma indutoides (P.D. Orton) Noordel.
22. Entoloma nigrovelutinum O.V. Morozova & A.V. Alexandrova
23. Entoloma rivulare Kokkonen
24. Entoloma undulatosporum Arnolds & Noordel.
25. Fasciodontia brasiliensis Yurchenko & Riebesehl
26. Fomitopsis marianiae (Bres.) Spirin, Vlasák & Cartabia
27. Geastrum argentinum Speg.
28. Geastrum fuscoglebum (Zeller) Jeppson & E. Larss.
29. Gymnopus cremeostipitatus Antonín, Ryoo & Ka
30. Gyroporus austrobrasiliensis A.C. Magnago & R.M. Silveira
31. Hebeloma affine A.H. Sm., V.S. Evenson & Mitchel
32. Hebeloma asperosporum Beker & U. Eberh.
33. Hebeloma flaccidum A.H. Sm., V.S. Evenson & Mitchel
34. Hebeloma neurophyllum G.F. Atk.
35. Helvella bicolor Raddi
36. Hemimycena mairei (E.-J. Gilbert) Singer
37. Inocybe amblyospora Kühner
38. Inocybe amelandica Bandini & B. Oertel
39. Inocybe bellidiana Bandini, B. Oertel & U. Eberh.
40. Inocybe bombina Bandini & B. Oertel
41. Inocybe castaneicolor A. La Rosa, Bizio, Saitta & Tedersoo
42. Inocybe dryadiana Bandini & B. Oertel
43. Inocybe lampetiana Bandini & B. Oertel
44. Inocybe minutissima Carteret & Reumaux
45. Inocybe morganae Bandini, B. Oertel & U. Eberh.
46. Inocybe perlucida Bandini & E. Ferrari
47. Inocybe plocamophora Singer, I.J.A. Aguiar & Ivory
48. Inocybe sambucella G.F. Atk.
49. Inocybe suryana Bandini & B. Oertel
50. Lactarius microbuccinatus H. Lee, Wisitr. & Y.W. Lim
51. Lactarius mitratus H. Lee, Wisitr. & Y.W. Lim
52. Lepiota andegavensis Mornand
53. Leucoagaricus roseilividus (Murrill) E. Ludw.
54. Leucocoprinus caeruleovertens (Justo, Bizzi & Angelini) M. Asif, Saba & Vellinga
55. Leucocoprinus pakistaniensis (S. Jabeen & A.N. Khalid) M. Asif & Saba
56. Leucocoprinus subvolvatus (Malençon & Bertault) M. Asif, Saba & Vellinga
57. Mallocybe leucothrix Matheny & M.E. Sm.
58. Mallocybe subtomentosa (Peck) Matheny & Kudzma
59. Marasmius occultatus Har. Takah.
60. Marasmius pallescens Murrill
61. Marasmius tangerinus Wannathes, Suwannar., Kumla & Lumyong
62. Mycena cicognanii Robich
63. Panus purpuratus G. Stev.
64. Peziza irina Quél.
65. Pholiotina calongei Siquier, E. Suárez, Salom & Planas
66. Pluteus castroae Justo & E.F. Malysheva
67. Pluteus decoloratus E. Horak
68. Pluteus fibrillosus Murrill
69. Pluteus inflatus Velen.
70. Pluteus siccus E.F. Malysheva
71. Pseudosperma amabile Bandini, B. Oertel & Wehr
72. Pseudosperma solare Bandini, B. Oertel & U. Eberh.
73. Ptychogaster rubescens Boud.
74. Ramaria paraconcolor Franchi & M. Marchetti
75. Rhodocybe formosa Vila, Contu, F. Caball. & A. Ortega
76. Rhodocybe griseonigrella (Vila, Contu, F. Caball. & Ribes) Vizzini, Vila, Picillo & Contu
77. Russula quercicola Razzaq, Shahid, Naseer & Khalid
78. Russula variicolor Murrill
79. Volvariella clavocystidiata Kapitonov & E.F. Malysheva

### 3.2. Diversity Comparison Across Various Ecological Types

#### 3.2.1. Dominant Families and Genera in Natural Ecological Environments (Protected Areas)

In natural ecological environments, a total of 31 dominant macrofungal families (≥10 species) were identified (Table 1), comprising 753 species in total. The major families include Agaricaceae, Inocybaceae, Russulaceae, Psathyrellaceae, and Entolomataceae. These dominant families represent 28.70% of all recorded families and account for 78.19% of the total number of species in natural ecological environments.

In natural ecological environments, 47 dominant genera of macrofungi (≥5 species) were identified (Table 1), comprising 590 species. The main genera include *Inocybe*, *Russula*, *Cortinarius*, *Entoloma*, and *Agaricus*. These dominant genera account for 17.34% of the total number of genera and 61.27% of the total number of species.

#### 3.2.2. Dominant Families and Genera in Urban Green Spaces and Parks

In urban green spaces and parks, a total of 10 dominant macrofungal families (≥10 species) were identified (Table 2), comprising 191 species. The main families include Agaricaceae, Psathyrellaceae, Polyporaceae, Inocybaceae, and Omphalotaceae. These dominant families represent 14.71% of all recorded families and account for 58.77% of the total number of species in urban green spaces and parks.

In urban green spaces and parks, 17 dominant genera of macrofungi (≥5 species) were identified (Table 2), comprising 137 species. The main genera include *Agaricus*, *Leucocoprinus*, *Candolleomyces*, *Entoloma*, and *Inocybe*. These dominant genera account for 11.81% of the total number of genera and 42.15% of the total number of species.

#### 3.2.3. Representative Shared Macrofungi in Different Ecological Types

A total of 249 macrofungal species were shared between natural ecological environments and urban green spaces and parks, belonging to 62 families and 121 genera, and accounting for 23.58% of all recorded species (Table 3). These shared taxa are primarily affiliated with families such as Agaricaceae, Psathyrellaceae, Polyporaceae, Inocybaceae, Omphalotaceae, and Pluteaceae, and with genera including *Agaricus*, *Leucocoprinus*, *Candolleomyces*, *Inocybe*, *Marasmius*, and *Trametes*. Most of these shared macrofungi are saprotrophic, capable of growing on a wide range of substrates, and are commonly found in forest soils, decaying wood, or grasslands.

**Figure 2:**
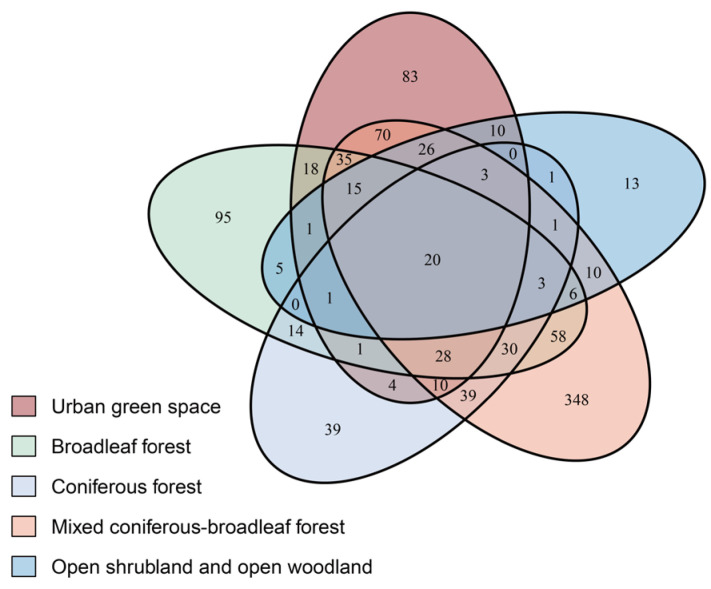
Venn diagram showing the overlap of macrofungal species among ecological types. The numbers in parentheses are the values of all observed fungi in each ecological type studied (cumulative species richness).

To illustrate the overlap of macrofungal species among different ecological types, a Venn diagram was constructed in this study, visually highlighting the shared and unique species within each type (Figure 2). The diagram reveals that many species are common to the four natural ecological types, with 20 species (such as *Coprinellus micaceus* (Bull.) Vilgalys, Hopple & Jacq. Johnson, *Schizophyllum commune* Fr., *Trametes hirsuta* (Wulfen) Lloyd, *Trametes versicolor* (L.) Lloyd) occurring in all of them, indicating their broad ecological adaptability.

#### 3.2.4. Representative Endemic Macrofungi

Representative endemic macrofungi in natural ecological environments (protected areas).

In natural ecological environments (protected areas), a total of 714 endemic macrofungal species were recorded, belonging to 96 families and 229 genera (Table 4). The dominant genera include *Inocybe*, *Russula*, *Cortinarius*, *Entoloma*, *Hebeloma*, *Amanita*, *Mycena*, *Lactarius*, *Pluteus*, *Geastrum*, *Tricholoma*, *Agaricus*, *Gymnopus*, *Helvella*, and *Marasmius*. Among these, ectomycorrhizal fungi represent the most characteristic group. These fungi typically form ectomycorrhizal symbiotic relationships with trees and are key components in maintaining forest health and supporting nutrient cycling in soils.

Representative endemic macrofungi in urban green spaces and parks.

In urban green spaces and parks, a total of 76 endemic macrofungi were recorded (Table 5), belonging to 29 families and 56 genera. The main genera include *Entoloma*, *Agaricus*, *Coprinopsis*, *Leucocoprinus*, *Calocybe*, *Cyathus*, *Leucoagaricus*, *Mallocybe*, *Marasmiellus*, *Pseudosperma*, and *Volvariella*. These unique taxa are predominantly saprotrophic fungi, playing important roles in organic matter decomposition and soil nutrient cycling.

## 4. Discussion

As an international metropolis, Beijing is home to a large number of parks and green cities, and the suburbs have important nature reserves such as Baihuashan National Natural Reserve. Through five years of large-scale fungal investigations in Beijing, we discovered that Beijing boasts astonishing species diversity: a total of 1056 macrofungi were recorded in Beijing, belonging to 2 phyla, 7 classes, 25 orders, 109 families, and 286 genera (Table S1). These include 12 new species, 456 species new records in Beijing, and 79 species new records in China. The results indicate that Beijing exhibits a high species diversity of macrofungi.

Among those species, many have economic value, including 116 edible species, 56 species that are both edible and medicinal, 123 medicinal species, and 58 poisonous species (Table S1). The wild species most consumed by residents include *Armillaria mellea* (Vahl) P. Kumm., *Suillus granulatus* (L.) Roussel, and others, while the main poisonous macrofungi include *Amanita oberwinkleriana* Zhu L. Yang & Yoshim. Doi, *Coprinellus micaceus* (Bull.) Vilgalys, Hopple & Jacq. Johnson, *Lepiota brunneoincarnata* Chodat & C. Martín, *Lysurus mokusin* (L.) Fr., and others [41,55].

Among the 1056 macrofungal species identified in Beijing, 542 species have been assessed for their threat status in the Redlist of China’s Biodiversity—Macrofungi, while 514 species remain unassessed (Table S1). The assessment results indicate that eight macrofungal species in Beijing require attention and conservation. Of these, species classified as Near Threatened include *Artomyces pyxidatus* (Pers.) Jülich, *Ganoderma applanatum* (Pers.) Pat., *Ganoderma sichuanense* J.D. Zhao & X.Q. Zhang, *Laccaria alba* Zhu L. Yang & Lan Wang, *Macrocybe gigantea* (Massee) Pegler & Lodge, *Ramaria stricta* (Pers.) Quél., and *Stropharia rugosoannulata* Farl. ex Murrill; those classified as Vulnerable include *Hericium erinaceus* (Bull.) Pers.; the remaining species are categorized as either Least Concern or Data Deficient [58,59].

Further analysis shows that the natural ecological environment (protected areas) comprises 31 dominant families and 47 dominant genera, with characteristic taxa primarily represented by *Inocybe*, *Russula*, *Cortinarius*, *Entoloma*, *Hebeloma*, *Mycena*, *Amanita*, *Lactarius*, *Geastrum*, *Pluteus*, *Tricholoma*, *Agaricus*, *Gymnopus*, *Helvella*, and *Marasmius*. Urban green spaces and parks include 10 dominant families and 17 dominant genera, with characteristic taxa mainly represented by *Entoloma*, *Agaricus*, *Coprinopsis*, *Leucocoprinus*, *Calocybe*, *Candolleomyces*, *Cyathus*, *Leucoagaricus*, *Mallocybe*, *Marasmiellus*, *Pseudosperma*, and *Volvariella*. The taxa shared between these two ecological types are primarily dominated by *Agaricus*, *Leucocoprinus*, *Candolleomyces*, *Inocybe*, *Marasmius*, and *Trametes*. These results indicated that the endemic taxa in natural ecological environments (protected areas) are primarily composed of ectomycorrhizal and saprotrophic fungi. This is mainly because natural ecological environments typically possess higher ecological integrity and greater vegetation diversity, especially abundant forest types and sufficient host plant resources, providing a diverse and stable symbiotic foundation for ectomycorrhizal fungi. Additionally, the richness of the soil’s organic matter and minimal disturbances in these environments favor the growth and differentiation of various saprotrophic fungi. Therefore, the rich vegetation structure and relatively stable soil microenvironment in natural ecosystems offer a favorable ecological basis for the symbiotic relationships of ectomycorrhizal fungi and the decomposition of organic matter by saprotrophic fungi [60,61]. The endemic taxa in urban green spaces and parks are primarily dominated by saprotrophic fungi, and the taxa shared between the two ecological types are also mainly saprotrophic. This is largely because urban green spaces and parks are predominantly artificial environments with limited plant diversity, often dominated by ornamental plants and lacking native forest trees. Consequently, the symbiotic plants required by ectomycorrhizal fungi are scarce, hindering the establishment of stable mycorrhizal associations. In contrast, saprotrophic fungi can directly utilize organic residues such as garden waste, fallen leaves, and wood chips as nutrient sources, and exhibit greater resilience to soil disturbances and microbial community changes typical of urban settings. Therefore, due to their low dependence on host plants and strong adaptability to anthropogenic disturbances, saprotrophic fungi become the dominant group in urban green spaces and parks, and serve as taxa commonly shared between natural and urban ecological types [62,63,64].

In the context of accelerating global biodiversity loss and intensifying climate change, it is crucial to deepen our understanding of and effectively protect macrofungal diversity, enhance public awareness and appreciation of the “Kingdom of Fungi”, and collaboratively foster a harmonious ecological vision of a “Beautiful Beijing” where humans and nature coexist. This is essential for advancing Beijing’s leading role in global urban ecological governance, supporting the national ecological civilization strategy, and contributing to the “Beijing Model” and “Chinese Wisdom”. This study provides fundamental data and a scientific basis for biodiversity conservation, as well as the development and utilization of macrofungal resources in Beijing.

## Figures and Tables

**Table 1:** Dominant families (≥10 species) and genera (≥5 species) of macrofungi in natural ecological environments.

Dominant Families				Dominant Genera		
Family	Number of Genera	Number of Species	Percentage (%)	Genera	Number of Species	Percentage (%)
Agaricaceae	12	81	8.41	*Inocybe*	50	5.19
Inocybaceae	4	67	6.96	*Russula*	41	4.26
Russulaceae	2	59	6.13	*Cortinarius*	27	2.80
Psathyrellaceae	8	45	4.67	*Entoloma*	27	2.80
Entolomataceae	3	35	3.63	*Agaricus*	25	2.60
Polyporaceae	16	32	3.32	*Amanita*	20	2.08
Cortinariaceae	2	28	2.91	*Geastrum*	18	1.87
Incertae sedis	12	28	2.91	*Hebeloma*	18	1.87
Pluteaceae	3	26	2.70	*Lactarius*	18	1.87
Omphalotaceae	4	24	2.49	*Marasmius*	18	1.87
Hymenogastraceae	4	23	2.39	*Pluteus*	18	1.87
Lycoperdaceae	5	23	2.39	*Mycena*	17	1.77
Amanitaceae	2	21	2.18	*Lepiota*	15	1.56
Marasmiaceae	3	20	2.08	*Leucocoprinus*	15	1.56
Mycenaceae	3	20	2.08	*Gymnopus*	14	1.45
Hymenochaetaceae	10	19	1.97	*Helvella*	13	1.35
Strophariaceae	5	19	1.97	*Melanoleuca*	12	1.25
Clitocybaceae	4	18	1.87	*Tricholoma*	12	1.25
Geastraceae	1	18	1.87	*Calocybe*	11	1.14
Boletaceae	11	17	1.77	*Lycoperdon*	11	1.14
Peniophoraceae	5	14	1.45	*Candolleomyces*	10	1.04
Physalacriaceae	5	14	1.45	*Conocybe*	10	1.04
Bolbitiaceae	4	13	1.35	*Leucoagaricus*	10	1.04
Helvellaceae	1	13	1.35	*Psathyrella*	10	1.04
Lyophyllaceae	2	12	1.25	*Trametes*	10	1.04
Tricholomataceae	1	12	1.25	*Collybiopsis*	9	0.93
Phanerochaetaceae	6	11	1.14	*Peniophora*	9	0.93
Schizoporaceae	4	11	1.14	*Mallocybe*	8	0.83
Hydnaceae	4	10	1.04	*Pseudosperma*	8	0.83
Irpicaceae	6	10	1.04	*Laccaria*	7	0.73
Phallaceae	4	10	1.04	*Parasola*	7	0.73
				*Pholiota*	7	0.73
				*Utraria*	7	0.73
				*Volvariella*	7	0.73
				*Clitocybe*	6	0.62
				*Collybia*	6	0.62
				*Coprinellus*	6	0.62
				*Coprinopsis*	6	0.62
				*Scleroderma*	6	0.62
				*Suillus*	6	0.62
				*Agrocybe*	5	0.52
				*Armillaria*	5	0.52
				*Clitopilus*	5	0.52
				*Hymenopellis*	5	0.52
				*Hypoxylon*	5	0.52
				*Stereum*	5	0.52
				*Xylodon*	5	0.52

**Table 2:** Dominant families (≥10 species) and genera (≥5 species) of macrofungi in urban green spaces and parks.

Dominant Families				Dominant Genera		
Family	Number of Genera	Number of Species	Percentage (%)	Genera	Number of Species	Percentage (%)
Agaricaceae	11	51	15.69	*Agaricus*	18	5.54
Psathyrellaceae	6	29	8.92	*Leucocoprinus*	13	4.00
Polyporaceae	12	22	6.77	*Candolleomyces*	9	2.77
Inocybaceae	4	18	5.54	*Entoloma*	9	2.77
Omphalotaceae	5	14	4.31	*Inocybe*	9	2.77
Pluteaceae	3	14	4.31	*Marasmius*	9	2.77
Entolomataceae	3	12	3.69	*Trametes*	9	2.77
Marasmiaceae	3	11	3.38	*Lepiota*	7	2.15
Incertae sedis	3	10	3.08	*Volvariella*	7	2.15
Russulaceae	2	10	3.08	*Collybiopsis*	6	1.85
				*Coprinellus*	6	1.85
				*Coprinopsis*	6	1.85
				*Geastrum*	6	1.85
				*Melanoleuca*	6	1.85
				*Pluteus*	6	1.85
				*Russula*	6	1.85
				*Calocybe*	5	1.54

**Table 3:** Number of families, genera, and species of shared macrofungi.

Family	Number of Genera	Number of Species	Percentage (%)
Agaricaceae	10	40	16.06
Psathyrellaceae	5	21	8.43
Polyporaceae	11	18	7.23
Inocybaceae	3	12	4.82
Omphalotaceae	4	10	4.02
Pluteaceae	2	10	4.02
Marasmiaceae	2	9	3.61
Phallaceae	4	9	3.61
Russulaceae	2	8	3.21
Incertae sedis	3	7	2.81
Geastraceae	1	6	2.41
Hymenochaetaceae	6	6	2.41
Entolomataceae	2	5	2.01
Peniophoraceae	4	5	2.01
Amanitaceae	2	4	1.61
Auriculariaceae	1	4	1.61
Irpicaceae	3	4	1.61
Lycoperdaceae	3	4	1.61
Bolbitiaceae	1	3	1.20
Lyophyllaceae	1	3	1.20
Peniophorellaceae	1	3	1.20
Phanerochaetaceae	3	3	1.20
Physalacriaceae	3	3	1.20
Sclerodermataceae	1	3	1.20
Strophariaceae	2	3	1.20
Bondarzewiaceae	1	2	0.80
Clitocybaceae	2	2	0.80
Fomitopsidaceae	1	2	0.80
Gloeophyllaceae	1	2	0.80
Helvellaceae	1	2	0.80
Hypoxylaceae	2	2	0.80
Oxyporaceae	1	2	0.80
Schizoporaceae	2	2	0.80
Tubariaceae	2	2	0.80
Boletaceae	1	1	0.40
Ceratobasidiaceae	1	1	0.40
Cerrenaceae	1	1	0.40
Clathraceae	1	1	0.40
Coniophoraceae	1	1	0.40
Dacrymycetaceae	1	1	0.40
Dacryobolaceae	1	1	0.40
Galeropsidaceae	1	1	0.40
Ganodermataceae	1	1	0.40
Hydnaceae	1	1	0.40
Hygrophoropsidaceae	1	1	0.40
Hyphodermataceae	1	1	0.40
Macrocystidiaceae	1	1	0.40
Meripilaceae	1	1	0.40
Meruliaceae	1	1	0.40
Mycenaceae	1	1	0.40
Paxillaceae	1	1	0.40
Pezizaceae	1	1	0.40
Pleurotaceae	1	1	0.40
Podoscyphaceae	1	1	0.40
Porotheleaceae	1	1	0.40
Postiaceae	1	1	0.40
Punctulariaceae	1	1	0.40
Schizophyllaceae	1	1	0.40
Steccherinaceae	1	1	0.40
Stereaceae	1	1	0.40
Suillaceae	1	1	0.40
Tapinellaceae	1	1	0.40

**Table 4:** Representative endemic macrofungi (≥5 species) in natural ecological environments (protected areas).

Genera	Number of Species	Percentage (%)
*Inocybe*	42	5.88
*Russula*	36	5.04
*Cortinarius*	27	3.78
*Entoloma*	24	3.36
*Hebeloma*	18	2.52
*Amanita*	17	2.38
*Mycena*	17	2.38
*Lactarius*	15	2.10
*Pluteus*	13	1.82
*Geastrum*	12	1.68
*Tricholoma*	12	1.68
*Agaricus*	11	1.54
*Gymnopus*	11	1.54
*Helvella*	11	1.54
*Marasmius*	10	1.40
*Lepiota*	9	1.26
*Lycoperdon*	9	1.26
*Calocybe*	8	1.12
*Leucoagaricus*	8	1.12
*Conocybe*	7	0.98
*Laccaria*	7	0.98
*Mallocybe*	7	0.98
*Melanoleuca*	7	0.98
*Parasola*	7	0.98
*Peniophora*	7	0.98
*Psathyrella*	7	0.98
*Utraria*	7	0.98
*Clitocybe*	6	0.84
*Collybia*	6	0.84
*Pholiota*	6	0.84
*Armillaria*	5	0.70
*Leucocoprinus*	5	0.70
*Pseudosperma*	5	0.70
*Stereum*	5	0.70
*Suillus*	5	0.70

**Table 5:** Endemic macrofungi in urban green spaces and parks.

Genera	Number of Species	Percentage (%)
*Entoloma*	6	7.89
*Agaricus*	4	5.26
*Coprinopsis*	4	5.26
*Leucocoprinus*	3	3.95
*Calocybe*	2	2.63
*Cyathus*	2	2.63
*Leucoagaricus*	2	2.63
*Mallocybe*	2	2.63
*Marasmiellus*	2	2.63
*Pseudosperma*	2	2.63
*Volvariella*	2	2.63
*Aleuria*	1	1.32
*Antrodia*	1	1.32
*Bovista*	1	1.32
*Calvatia*	1	1.32
*Candolleomyces*	1	1.32
*Chamaemyces*	1	1.32
*Collybiopsis*	1	1.32
*Coniophora*	1	1.32
*Conocybe*	1	1.32
*Crinipellis*	1	1.32
*Efibula*	1	1.32
*Entocybe*	1	1.32
*Flammulina*	1	1.32
*Fomitopsis*	1	1.32
*Ganoderma*	1	1.32
*Henningsomyces*	1	1.32
*Hyphodontia*	1	1.32
*Inocybe*	1	1.32
*Kuehneromyces*	1	1.32
*Laccaria*	1	1.32
*Lacrymaria*	1	1.32
*Lactarius*	1	1.32
*Lenzitopsis*	1	1.32
*Lepiota*	1	1.32
*Marasmius*	1	1.32
*Mattirolomyces*	1	1.32
*Melanoleuca*	1	1.32
*Mycetinis*	1	1.32
*Neolentinus*	1	1.32
*Panus*	1	1.32
*Parasola*	1	1.32
*Pilatoporus*	1	1.32
*Pluteus*	1	1.32
*Polyporus*	1	1.32
*Psathyrella*	1	1.32
*Pseudocercospora*	1	1.32
*Pseudosperma*	1	1.32
*Russula*	1	1.32
*Scleroderma*	1	1.32
*Sinoperdon*	1	1.32
*Stropharia*	1	1.32
*Trametes*	1	1.32
*Vanderbylia*	1	1.32
*Volvopluteus*	1	1.32
*Xanthoperenniporia*	1	1.32

## Data Availability

The data presented in this study are openly available in the Mycological Herbarium, Institute of Microbiology, Chinese Academy of Sciences, Beijing, China (https://nmdc.cn/fungarium/fungi/chinastrain, accessed on 27 June 2025).

## Supplementary Materials

The following supporting information can be downloaded at https://www.mdpi.com/article/10.3390/jof11080607/s1: Table S1: Checklist of macrofungi in Beijing.
